# A Method for Digital Color Analysis of Spalted Wood Using Scion Image Software

**DOI:** 10.3390/ma2010062

**Published:** 2009-02-16

**Authors:** Sara C. Robinson, Peter E. Laks, Ethan J. Turnquist

**Affiliations:** Michigan Technological University / 1400 Townsend Dr., Houghton, MI 49931, USA; E-Mails: plaks@mtu.edu (P.L.); ejturnqu@mtu.edu (E.T.)

**Keywords:** **Keywords**: Spalting, Scion Image, color analysis.

## Abstract

Color analysis of spalted wood surfaces requires a non-subjective, repeatable method for determining percent of pigmentation on the wood surface. Previously published methods used human visual perception with a square grid overlay to determine the percent of surface pigmentation. Our new method uses Scion Image^©^, a graphical software program used for grayscale and color analysis, to separate fungal pigments from the wood background.  These human interface processes render the wood block into HSV (hue, saturation, value, within the RGB color space), allowing subtle and drastic color changes to be visualized, selected and analyzed by the software. Analysis with Scion Image^©^ allows for a faster, less subjective, and easily repeatable procedure that is superior to simple human visual perception.

## 1. Introduction

Spalting is a natural coloration process in which fungi with pigmented hyphae colonize wood in a high enough concentration for a visual, macroscopic color change to occur. Zone lines, areas of melanized hyphal tissue or pseudosclerotial plates, and the bleaching effect caused by many white rot fungi are also considered to be spalting [1]. Spalted wood has been used for decorative purposes for hundreds of years. Examples range from the Intarsia inlay of the 14^th^ and 15^th^ centuries to modern day wood sculptures [2]. Recent consumer trends have brought spalted wood out of the purely artistic world and into the consumer domain, resulting in many ‘home spalting recipes’ [3]. Increased interest from commercial businesses looking to produce and market spalted wood [4] has lead to an interest in inducing spalting under controlled, repeatable conditions [1]. 

Inducing and stimulating pigment responses in fungi requires quantitative measurement techniques for accurately reporting results. Consumer preference varies, but in general there is a preference for heavily spalted wood with many zone lines. Wood that is more heavily spalted will fetch a higher price than lightly spalted wood. Due to this, it is necessary to have a method to evaluate the amount of spalting on a given piece of wood. Previous research with spalted wood led us to develop a test for amount of surface spalting [1], which was based entirely on human perception. A clear piece of acetate (37 mm x 37 mm) was divided into 16 equal parts and overlaid onto one face of each 37 mm spalted test block. The number of squares which contained pigmentation, bleaching, and/or zone lines were recorded and divided by 16 to determine the percent of each type of spalting. This method, while quick, was limited by the researcher’s visual color perception and ability to distinguish areas of spalting on small test blocks. Overhead lighting, amount of daylight, and changes in air humidity all affected the acetate grid results by changing the visual perception of color on the wood. In addition, blocks could not be reanalyzed at a later date due to color changes over time. 

Several methods exist for measuring color in wood using human-machine interfaces. A traditional approach uses tristimulus colorimeters to measure the absorbance of different light wavelengths [5]. The advent of digital image analysis has created rapid, less expensive analysis methods, and can be used for a wider range of applications such as distinguishing compression wood from “normal” wood [6]. Digital imaging software has been used for several years to analyze various color elements. Adobe Photoshop^©^ is used for analysis of chromogen signals by using the eyedropper tool to select and highlight colors of interest. The number of pixels highlighted is then analyzed using the Histogram tool [7]. NIH Image^©^, a public domain software package, is used to quantify immunocytochemical data by highlighting density slices of interest that have been sliced from the background [8]. CIE Lab^©^ software can be used to analyze colors from digital photographs using the RGB system [9]. While all of these software packages allow for isolation and analysis of colored areas, none provide a quick method for multiple color analysis combined with a total surface area analysis.

Scion Image^©^ is a digital graphic analysis program that is used in many fields. It can be used for digital photo layers, such as described by Stuurman *et al*. [10], who created 3-D projections from photos of plant roots inoculated with bacteria. However, Scion Image^©^ is primarily used with biological systems as a method for frequency counts, or measurement of the area covered by a specific organism. For example, Allaire *et al*. [11] used Scion Image^©^ to detect the total number of nuclei within samples of animal tissue by manually assessing color differences within the image. Hopp and Smausz [12] used Scion Image^©^ to determine the amount of germinated conidia covering their culture mediums within a grayscale image. Although the program was not originally intended for color analysis, Tolivia *et al*. [13] used Scion Image^©^, along with Adobe Photoshop^©^, to quantify signals in histochemistry, while the USDA Forest Service’s Northern Research Station uses Scion Image^©^ for red and green leaf color analysis [14]. 

Color analysis of spalted wood brings together several aspects from the aforementioned literature. A total count (total area covered by spalting) is required, however the count cannot be monochromatic. Instead, it is necessary to read and analyze varying shades of the interest color, such as with zone lines that can vary in hue from green to black, or to analyze several different colors within the same space, as with fungi that are capable of multiple types of spalting. It is also necessary for the program to recognize and separate spalting from the wood’s natural color variations, and to be able to ‘subtract’ unwanted color, such as from residual fungal mycelium or culture medium. A method is still needed for full color analysis of digital images despite the frequent use of Scion Image^©^ in current research. A preferable method would be one in which the HSV 3-D model is used to select areas of interest for analysis. In addition, a method would be needed to analyze true black, which cannot be analyzed in an HSV model within Scion Image^©^. 

Our objectives were to combine previously published color analysis techniques with the modified Murakami *et al.* [14] leaf color analysis to create a repeatable method for color analysis of spalted wood. Previously published color analysis techniques cannot be used with spalted wood due to spalted wood’s frequent low saturation colors, shade and color changes within the same type of spalting, and the need for a total background area count. We also required methods to measure pure black, which cannot be measured with the full spectrum LUT tool in Scion Image^©^, and a method for increasing low color contrasts that are undifferentiated by Scion Image^©^. Developing a reliable and repeatable method of color analysis for spalted wood is necessary for reporting induced spalted results from both laboratory evaluations and commercial development.

## 2. Results and Discussion

The spalting results for four fungi are summarized in Table 1. All inoculated blocks were spalted by the end of 10 weeks (8 weeks for *T. versicolor*). *Trametes versicolor* produced bleaching and light black spotting on inoculated blocks, while *Xylaria polymorpha* produced heavy, dark zone lines and light bleaching. *Ceratocystis virescens* and *Arthrographis cuboidea* produced a low-saturation bluestain. Uninoculated control blocks were generally darker than before incubation due to abiotic hemicellulose hydrolysis and phenolic compound oxidation [15], as well as movement of water-soluble compounds from the center of the blocks to the outside surfaces [16].

**Table 1:** Mean percent external spalting area for four fungi. Numbers in parenthesis indicate standard deviation. N=5 replicates.

Fungus	Bleaching	Zone Lines	Bluestain
*T. versicolor*	47.33 (8.30)	2.66 (1.49)	-
*X. polymorpha*	9.63 (6.73)	28.89 (16.80)	-
*A. cuboidea*	-	-	21.59 (27.93)
*C. virescens*	-	-	10.17 (7.90)

Bleaching occurred on approximately 47% (SD: 8.3%) of the external surface of blocks inoculated with *T. versicolor* (Table 1)*.* Zone lines covered only 2.66% (SD: 1.49%) of the surface of these blocks. Scion Image^©^ easily detected the bleached areas due to the darkening of the rest of the block by water contact and drying. The zone lines appeared as faint brown/black dots, and were analyzed with a color analysis due to the absence of true black. 

The bleaching and the zone line analyses were less subjective than visual analysis. However, subjectivity still existed, as Scion Image^©^ still required the user to manually maneuver the LUT bar to cover the spalted areas. This action requires visual perception of where the spalting occurred on the original block image. The potential variability of these results is significantly decreased if only one user is responsible for doing all analyses within a short timeframe. Long breaks from analysis (such as over a weekend) could lead to the user ‘resetting’ their definition of how strong the spalting must appear in order to be measured, in effect creating two sets of data.

*Xylaria polymorpha* produced heavy black zone lines (28.89% SD: 16.80%) and light bleaching (9.63% SD: 6.73%). The zone lines contained both true black and varying lighter shades of black and brown. The combination of the true black analysis and the color analysis captured all of the zone line areas, without picking up the darker areas of the wood block. 

The true black analysis proved to be the easiest and least subjective of the measurements, as it did not require user input. Every block was analyzed under the same color specifications by placing the LUT tool completely to the bottom of the white bar. The addition of lighter blacks and browns analysis via the color analysis Result* window introduced some subjectivity, but the results were still far less subjective than just a Result* color analysis.

Both *Ceratocystis virescens* and *Arthrographis cuboidea* produced a low-saturation bluestain (10.17% SD: 7.90%, and 21.59% SD: 27.93%, respectively). All of these blocks were edited in Photoshop^©^ to increase their contrast before being analyzed with Scion Image^©^. After editing, the bluestain appeared as true black and occasionally as a very dark red. These colors were then analyzed using the basic color analysis method, and added together to give a total bluestain amount. The Photoshop^©^-edited images became the easiest images to analyze due to the easy and repeatable true black analysis. Thus, the bluestain percentages were the most consistent and least subjective of all the measurements.

It would be possible to use Photoshop^©^ to convert all colors of interest into true black to make the analysis readings less subjective. However one of the large drawbacks of increasing contrast in Photoshop^©^ is the inability to select which hues are increasing in contrast. With the bluestain blocks, all darker areas became true black. This meant that block surface areas that had saw kerf gouges or staining also became true black. These areas had to be sanded smooth and scanned again before analysis. In addition, a multiple color analysis could not be performed on images converted in Photoshop^©^, as all the darker colors would be combined into the true black. Photoshop^©^ is recommended for conversion in instances of a single color analysis where saturation is too low to be read by Scion Image^©^. 

A plug-in is available for Photoshop^©^ that can facilitate the aforementioned conversion. The Color Deconvolution can separate a color of interest from both one other color, and the background color [17]. This software has been used successfully for evaluation of immunohistochemical staining [18]. The user can select a low saturation color to be distinguished from the surrounding area by inverting the colors, causing the color of interest to stand out against the background. This plug-in provides a more precise selection of colors for conversion of low saturation images. However, the free trial version of the software cannot analyze images over 250 pixels or 8 bit resolution and a donation must be made to access the full version of the software.

Using either original or color converted images with Scion Image^©^ provides a superior color analysis when compared with current procedures. Previously published color analysis methods should not be used to evaluate spalted wood. For example, Lehr *et al.* [7] used Photoshop^©^ to highlight, select, and analyze colored pixel amounts using the eyedropper tool and the Histogram function. This number can be divided by the total pixel amount to determine a color percentage. This analysis method is limited, as the magic wand color tolerance often does not cover the wide color gradients produced in spalted wood. Highly colonized areas of spalted wood contain higher saturations of color than areas with less colonization. Due to colonization differences, spalting produced by some fungi may contain saturation and color changes. These different saturations and colors must be analyzed separately with Adobe Photoshop^©^ due to the limitations of the threshold range of the eyedropper tool. This results in overlapping measurements and inflated color amounts. The LUT tool in Scion Image^©^ allows for an infinite tolerance for each analyzed color, which results in fewer measurements and no chance of colored areas being measured twice.

Colors in wood can also be analyzed using a combination of the CIELAB color scale and either Adobe Photoshop^©^ or Corel Photo Paint^©^ [9]. CIELAB allows the user to compare values for L (white/black), a (red/green), and b (yellow/blue) of a ‘standard’ piece of wood (such as one with no spalting) to the values produced from a piece with spalting. The total color difference can be calculated, however the program cannot provide information on which colors are different. This makes multiple color analysis impossible.

Tolivia *et al.* [13] used Adobe Photoshop^©^ and Scion Image^©^ for color signal strength. This method uses the color range tool instead of the eyedropper tool to allow tolerance additions or subtractions to be viewed in real time. The selected color is cut from the original image, pasted to a blank template, and changed to grayscale. The strength of the grayscale signal is analyzed in Scion Image^©^ using Mean Density tool. This method of analysis cannot be altered for color analysis of spalted wood as there is no possibility for a complete area count of the original block. 

Murakami *et al.* [14] used only Scion Image^©^ for color analysis of leaves. Our color analysis used the same image math procedure as outlined in Murakami *et al* but expanded on the types of analyses that could be performed. Analysis of true black, true white, multiple colors, and low saturation colors were added to the Murakami procedure to allow for the complexities of color analyzing spalted wood.

## 3. Experimental Section 

### 3.1. Spalting procedure

Four known spalting fungi were selected for use in this experiment based upon the type of spalting they produced. *Trametes versicolor* (L:Fr.) Pilat (MAD 697) is a quick-growing white rot fungus of hardwoods. It produces both external and internal bleaching in sugar maple blocks by 6 weeks of incubation, and is one of the few fungi capable of producing zone lines without external antagonism [1]. *Xylaria polymorpha* (Pers.) Grev. (SR001) causes moderate to light white rot in hardwoods and is a prolific zone line producing fungus [19]. *Arthrographis cuboidea* (Sacc. & Ellis) Sigler (DR451) causes pink stain in hardwoods, although research has shown that it can also produce bluestain [20]. *Ceratocystis virescens* (R.W. Davidson) C. Moreau (C252) is a common bluestain of sugar maple.

We obtained the fungi from the following sources:*Trametes versicolor* from the Forest Products Laboratory in Madison Wisconsin, *Xylaria polymorpha* (SR001) from a sugar maple stump in Alberta, MI (collected by S.C. Robinson), *Arthrographis cuboidea* from a southern yellow pine board originating from Memphis, TN (collected by Dr. D.L. Richter) and *Ceratocystis virescens* from Dr. T. Harrington Mantle, NY (collected by D. Houston). 

Clear, unstained sugar maple wood was cut into 14 mm cubes and oven dried at 40°C for 24 hours before inoculation. Blocks were free of stain, knots, and other color deviations. The sugar maple was obtained in Houghton Co., Michigan by hand felling. It had a green specific gravity of 0.65 and a density of 742 kg m^-3^ at 12% moisture content.

The fungi were inoculated onto the sugar maple blocks and incubated in square flint jars (4x4x12 cm) containing 50 mL of distilled water and 15 grams of medium-size premium grade vermiculite (Strong-Lite Products Corp^©^). The jars were prepared based on the procedure outlined in Robinson *et al*. [21], and with a water content equal to 98% of the vermiculite water holding capacity (WHC). A total of 25 jars were set up; five for each fungus and five to serve as uninoculated controls. All jars were autoclaved for 35 minutes, and the blocks steam sterilized for 30 minutes before inoculation. Blocks inoculated with *X. polymorpha, A. cuboidea,* and *C. virescens* were incubated for 10 weeks in the dark in an incubation room (27°C ± 2°C, 80% ± 5% relative humidity). Blocks inoculated with *T. versicolor* were incubated for 8 weeks.

### 3.2. Color analysis – general procedure

Blocks were removed from the jars after their incubation time, rinsed with tap water, and then scrubbed with a soft bristle brush to remove vermiculite and mycelium. Blocks had to be completely cleaned of all foreign material before scanning to insure only the block surface was analyzed by Scion Image^©^. After cleaning, the blocks were dried overnight in a forced air dryer at 40°C. Dried blocks were scrubbed again before scanning to remove any loose wood fibers or dried mycelium. 

The dry blocks were scanned at 2400 dpi using an Epson Perfection V100 Photo Scanner. The transverse face with the most spalting was scanned and analyzed for pigmentation (blue, brown, white) and true black and true white using the procedure outlined below. The procedure is based on the leaf color analysis method of Murakami *et al.* [14].

Each .tif file was analyzed separately, however, all the scans from each fungus type were analyzed within two days of each other, and not in tandem with any other color analyses. This reduced the likelihood of ‘resetting’ the researcher’s definition of the color to be analyzed by comparing results from one fungus to another. This precaution was especially important when analyzing bleaching, as wood bleached by one fungus can look drastically different from bleaching produced by another fungus.

**Figure 1:**
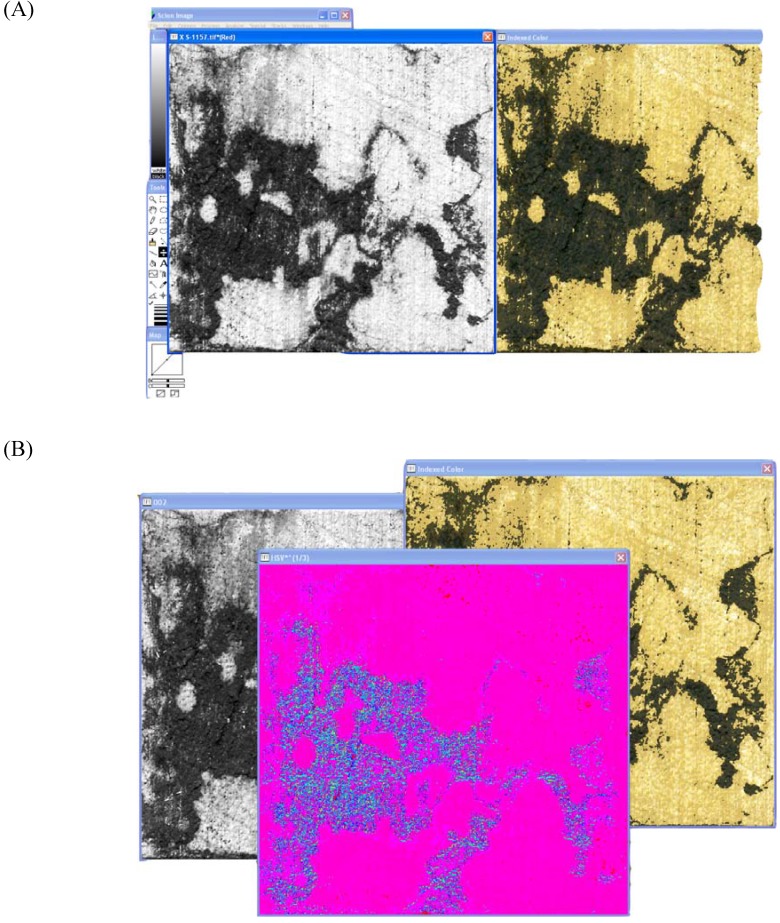

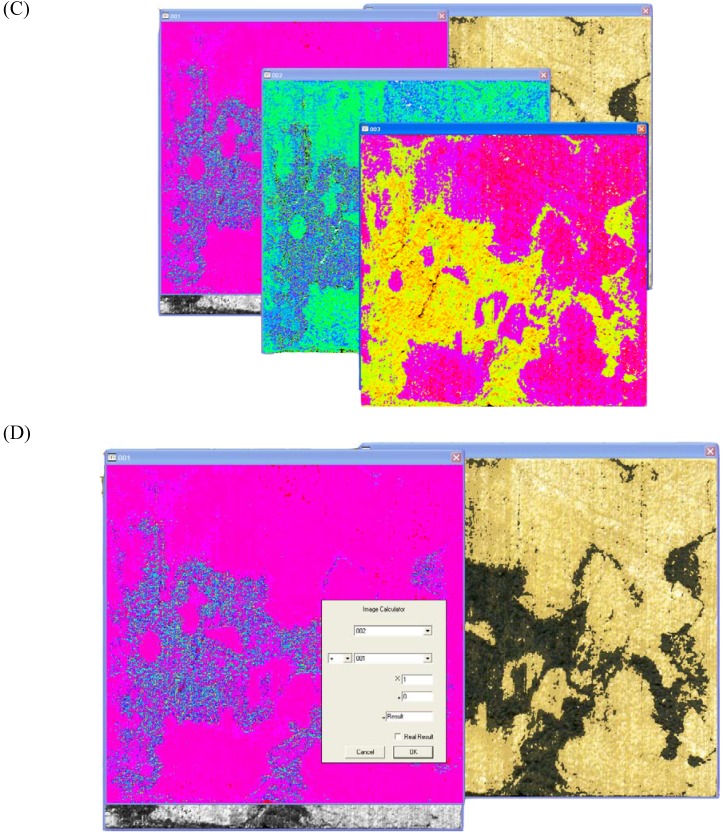
Image preparation for color analysis in Scion Image^©^. (A): The black and white and original color windows that appear in Scion Image^©^ after loading an image. (B): RGB to HSV conversion on the color image. (C): HSV image stacked to windows. (D): Image math, which adds image 2 (b/w) to image 1 (HSV).

Scion Image^©^ produces the original color, and also a grayscale copy after loading the file (Figure 1A). The grayscale copy is ‘stacked to windows’, which gives three versions of the original grayscale image. The version with the preferred brightness level is selected (usually version 2), and the remaining grayscale images are closed. The color image is then selected and the 8-bit color image is transformed into the RGB color space. The new image appears in its own window and is transformed into HSV (Figure 1B). 

Once in HSV, the image is again ‘stacked to windows’ (Figure 1C). This time the three windows are distinctly different - green/blue, magenta/blue and magenta/red versions of the HSV image. The magenta/red image is kept while the other two windows are closed. This image titled ‘001’ is ‘added’ to the grayscale image ‘002’ under image math. The addition field set to 0 and the multiplication field set to 1 (Figure 1D). 

**Figure 2:**
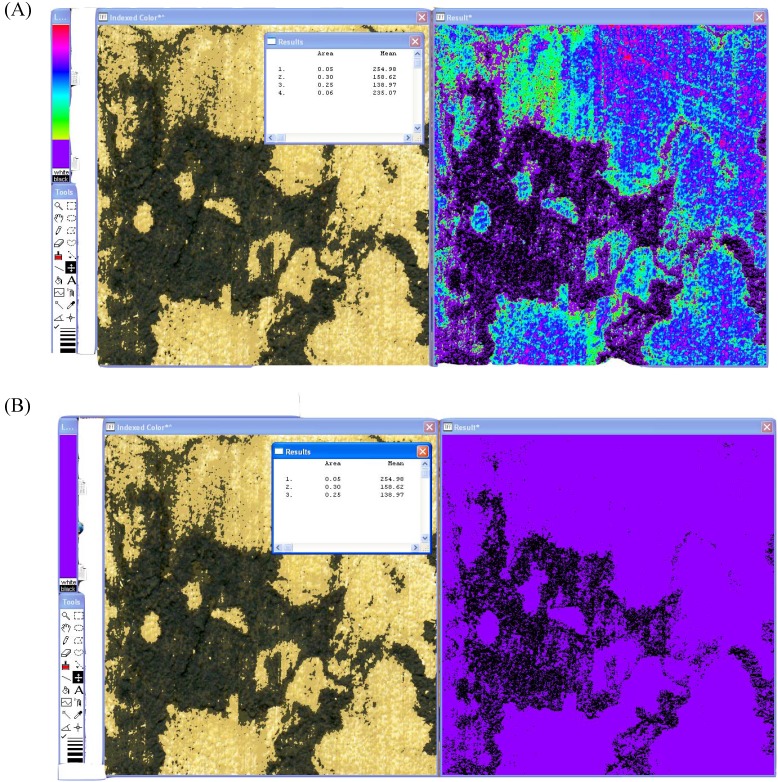
Color analysis of spalted wood using the LUT tool. (A): Analysis of zone line pigments except true black from the HSV image. (B): Total area measurement of the HSV image.

The ‘Result*’ image can then be compared to the original 8-bit color image for the spalting analysis. Every major and subtle color change within the original image is mirrored in the ‘Result*’ image in bright, contrasting colors. The LUT tool is then used to cover the parts of the spectrum on the ‘Result*’ image that correspond to the spalted areas of interest. Once the area of interest is covered, the image is analyzed using the ‘Analyze – Measure’ option (Figure 2A). A second analysis is performed by using the LUT tool to cover the entire spectrum (Figure 2B). The original analysis number is divided by the second, total area number, to give percent spalting. Standard deviation of the mean percent is then calculated using Microsoft Excel. 

Lower saturation spalting was not always immediately apparent. In these instances, fungus-inoculated blocks were compared with the uninoculated controls for a color comparison. This comparison allowed the spalting color change to be differentiated from unrelated color changes in the wood due to drying and water-soluble compound movement.

### 3.3. Color Analysis – True black

The standard procedure outlined above can analyze all colors except true black, which is excluded from both the spalting and total analysis. In order to measure true black, such as the black produced by *X. polymorpha* zone lines, the ‘Result*’ image is transformed into a threshold image. 

**Figure 3:**
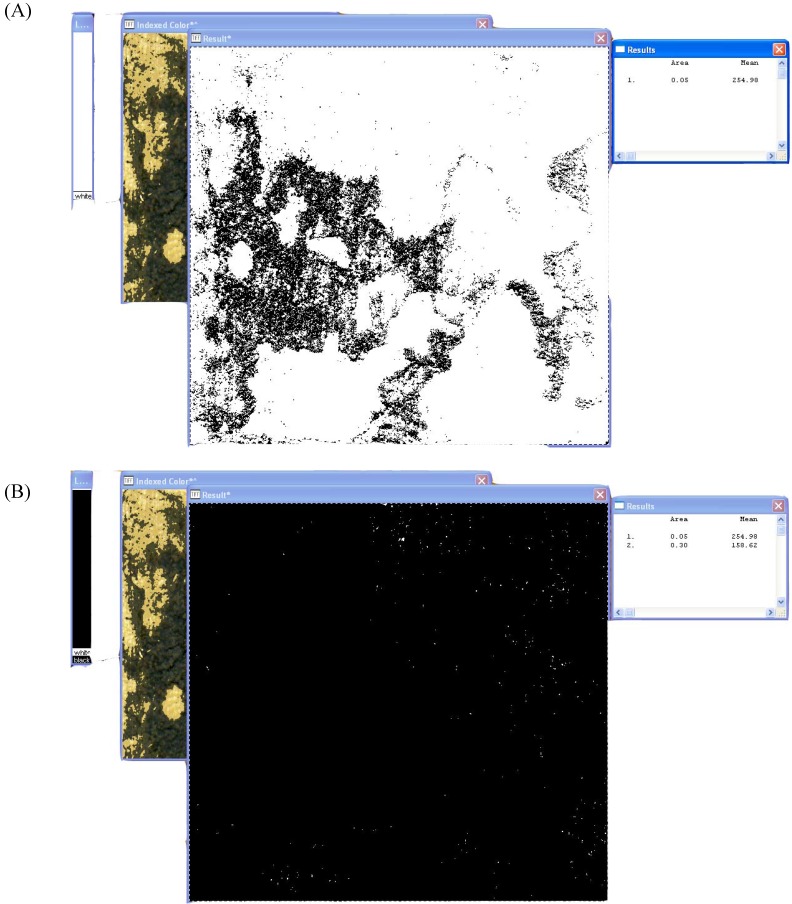
Analysis of true black using threshold and the LUT tool. (A): True black analysis only. (B): Total area analysis.

This produces a new scale that ranges only within white. The LUT tool is moved completely to the bottom and the image is analyzed (Figure 3A). The number produced represents the amount of true black within the image. When true black exists, the LUT tool must be moved to cover the entire white bar. A second reading is then taken to determine the total area (Figure 3B). The total area measurement produced by the general color analysis, as outlined above, will not be accurate if true black is present, as the procedure does not include true black in the measurement.

### 3.4. Color Analysis – True white and bleaching

Bleaching is analyzed by covering the top section of the color scale with the LUT tool. Usually only the lighter reds and purples will need to be covered to give an accurate bleaching assessment. The procedure is nearly the same as outlined in the General Procedure section, however a subtraction of true white must be made to account for white produced from remaining vermiculite or mycelial clumps. Blocks that were not sufficiently cleaned of mycelium often have small clumps remaining on their scanned surface. These clumps may not be visible to the naked eye but can appear as large white dots on the image after scanning. Blocks may also have small pieces of vermiculite still attached. These pieces of vermiculite reflect the scanner light and appear white on the digital image. If the mycelium or vermiculite are on bleached areas they will not skew the bleaching analysis; however, Scion Image^©^ will read them as bleaching if they are within a normal wood pigment area.

An extra reading must be taken to address this issue. The LUT tool must be moved to cover the entire area of the white bar under the threshold option. This reading gives a complete area minus true white, which is the color reading given by most mycelium and vermiculite. This number, subtracted from the total area from the Result* window color analysis, will give the total amount of true white. The amount of true white must be subtracted from the bleaching amount before that number is divided by the threshold total. Equation 1 illustrates the procedure:

((tt-ct)-bt)/tt=abt
(1)

where tt=threshold total, ct=total area under Result* color view, bt=bleaching total from the Result* color view, abt=actual bleaching total.

### 3.5. Color analysis – multiple colors

Multiple colors can be analyzed by taking two or more readings with the LUT tool covering only one color at a time. The separate color readings are then divided by the same overall reading as described above.

**Figure 4:**
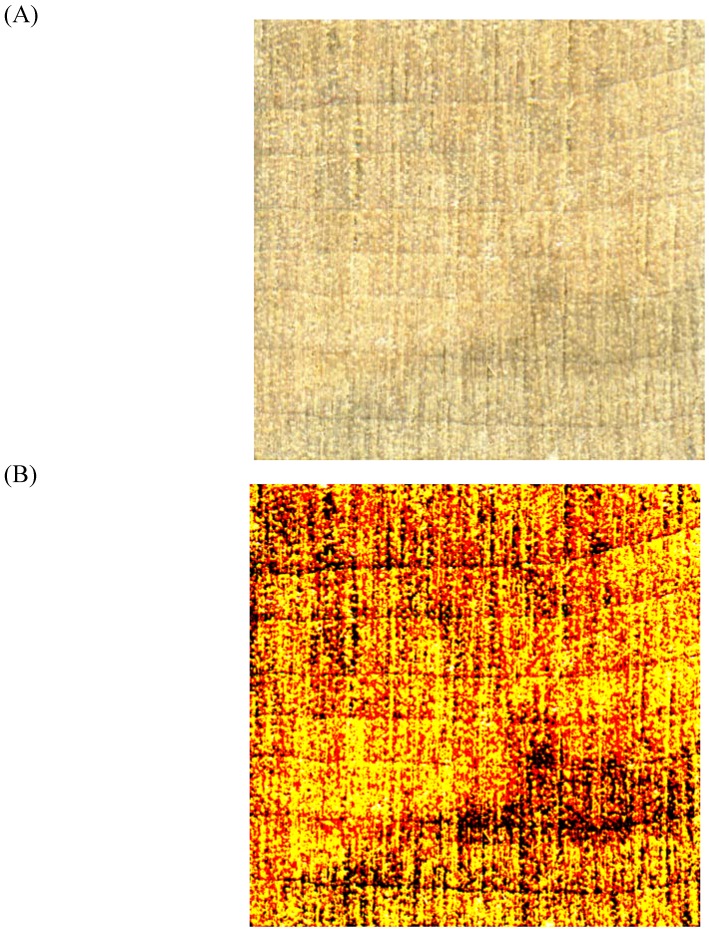
Original scan (A) and increased saturation scan (B) of an *Arthrographis cuboidea* inoculated block. Blue pigments have been saturated to true black and dark red.

If two or more of the colors of interest have similar saturations, or the fungal pigment has the same saturation as the wood, the scanner may be unable to adequately capture the color differentiation. Scion Image^©^ will not be able to separate the colors within the Result* image if the color difference cannot be easily seen on the scanned image. The saturation levels must be artificially increased in an image editor before analysis can occur. For example, low-saturation bluestain can be turned to true black and red in Photoshop^©^ by increasing the contrast under ‘image/adjustments’ (Figure 4). The true black and darker red areas can be read separately by Scion Image^©^, and then added together to give a total bluestain amount.

## 4. Conclusions 

The goal of this research was to manipulate digital scans in Scion Image^©^ to select and analyze areas containing any pigment, including true black and white. Our approach to color analysis of spalted wood used the basic Image Math processes as outlined by Murakami *et al*. [14], with specific modifications for subtle color differentiation, analysis of true black, and subtraction of true white. This modified process allows us to detect varying levels of bleaching (light to heavy), variations within zone line pigmentation (true black to brown), and a range of colored pigmentation (blue, etc). It also allows subtraction of unwanted colors to obtain more accurate results.

Low-saturation pigments were converted to true black in Photoshop^©^ before being analyzed in Scion Image^©^ to allow for greater accuracy and less subjectivity than Scion Image^©^ alone. In general, the use of Scion Image^©^ provides a quick, repeatable method for the digital analysis of spalting amounts on wood surfaces. This procedure is less subjective than an acetate grid overlay, requires less expensive equipment than a tristimulus colorimeter, and provides a comprehensive digital method of wood color evaluation that was not possible with previous analysis techniques.
